# Supplementary material for: Bio-economic indicators of fisheries: impact of variations in landings and fish size on market prices in Istanbul Fish Market
Source: PeerJ. 2023 Apr 4;11:e15141. doi: 10.7717/peerj.15141 (PMC10081457; doi:10.7717/peerj.15141)
Supplement: Supplemental Information 1 [file peerj-11-15141-s001.docx]

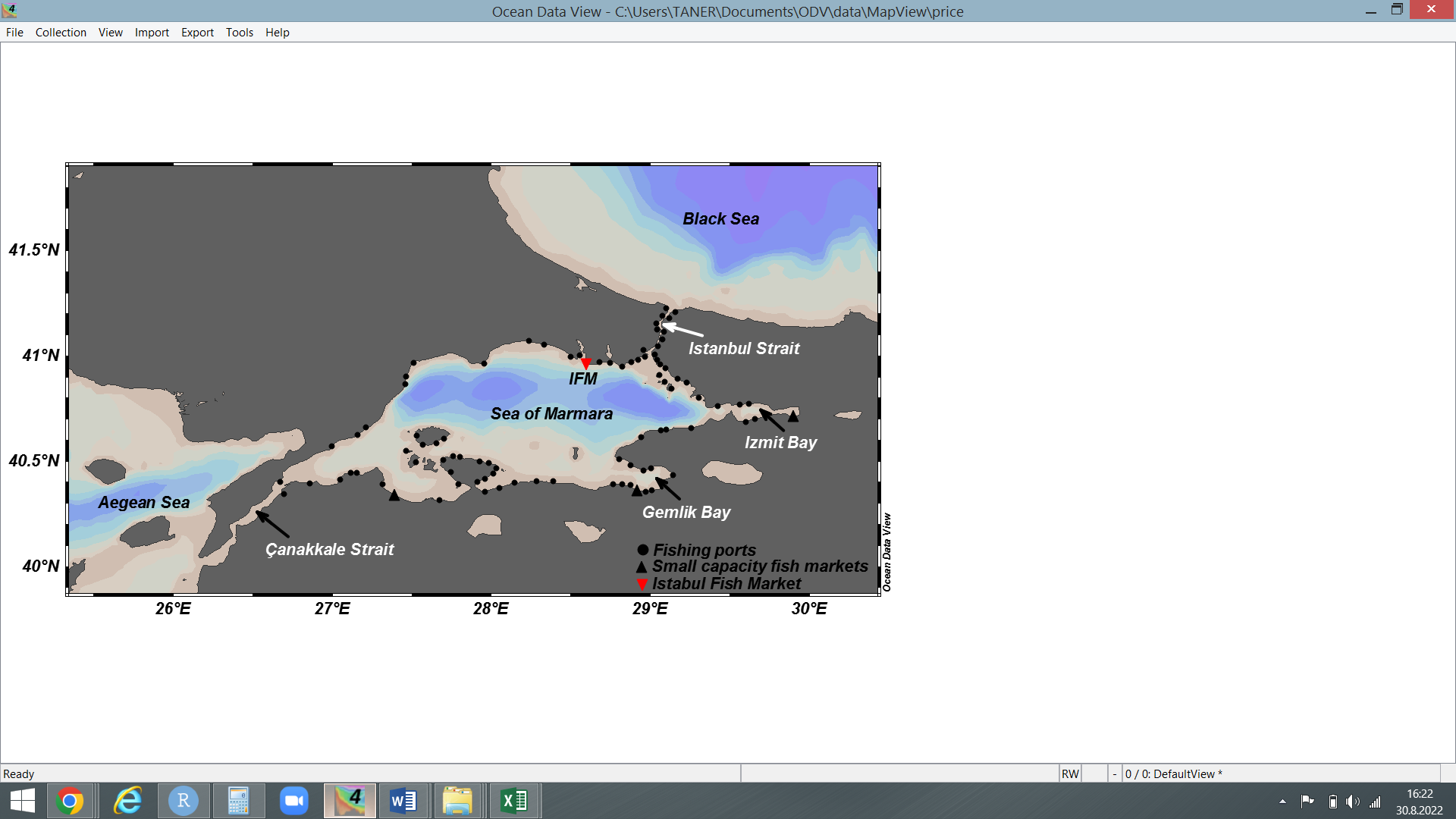


**Figure S1.** The study area, Sea of Marmara and location of Istanbul Fish Market (red triangle) together with fishing ports (black dots) and small capacity fish markets (black triangle).


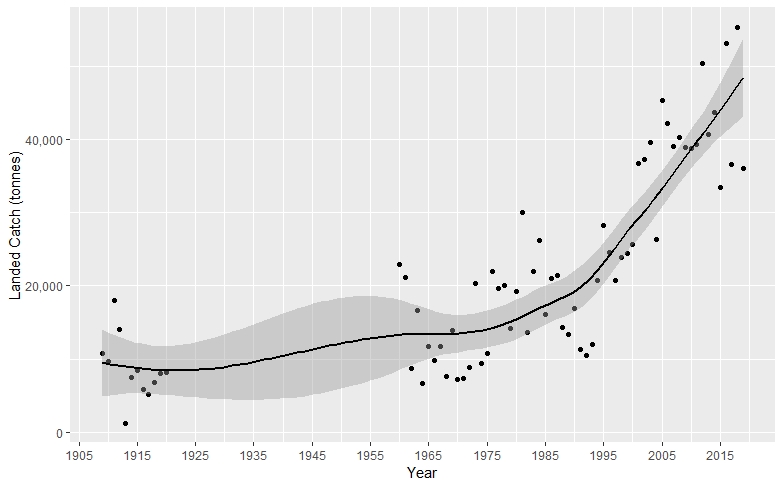


**Figure S2.** Long term landed catch quantity in the IFM


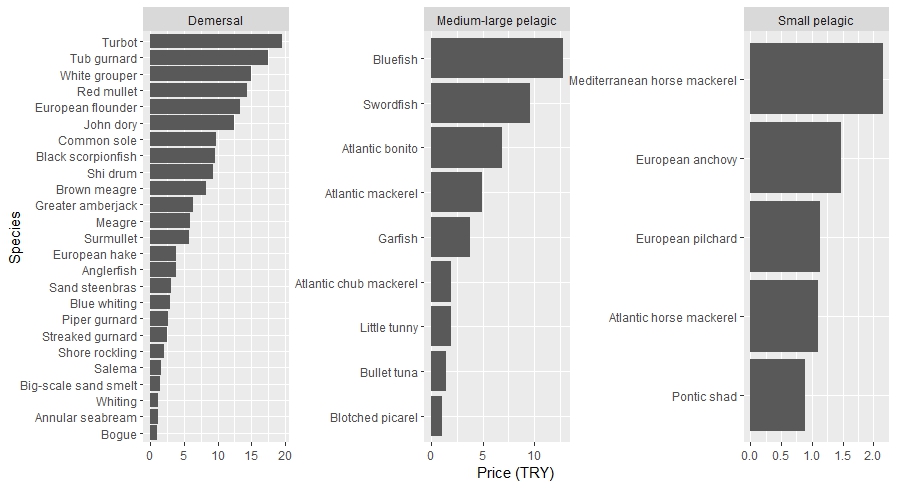


**Figure S3.** Mean prices (per unit/Turkish Lira, averaged from 2006-2019) for groupings of studied species, for A) demersals, B) pelagics, and C) small pelagics.


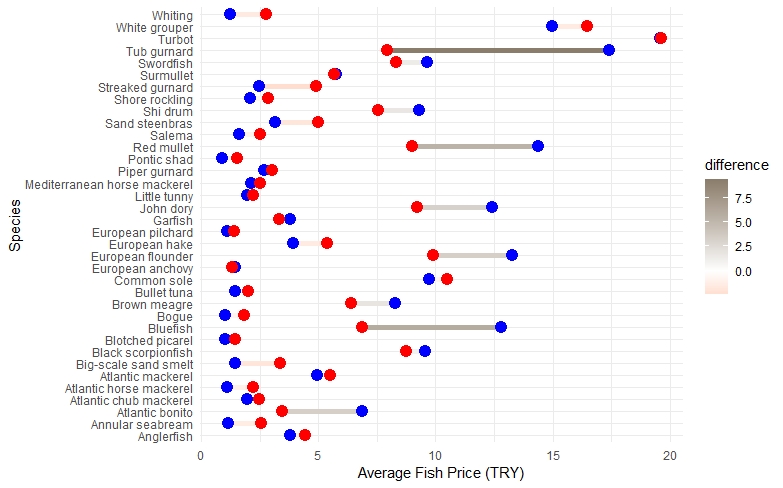


**Figure S4**. Mean price (2006-2019) comparison between Istanbul Fish Market and Turkish general (Red and blue points indicate the Turkish general and IFM, respectively)


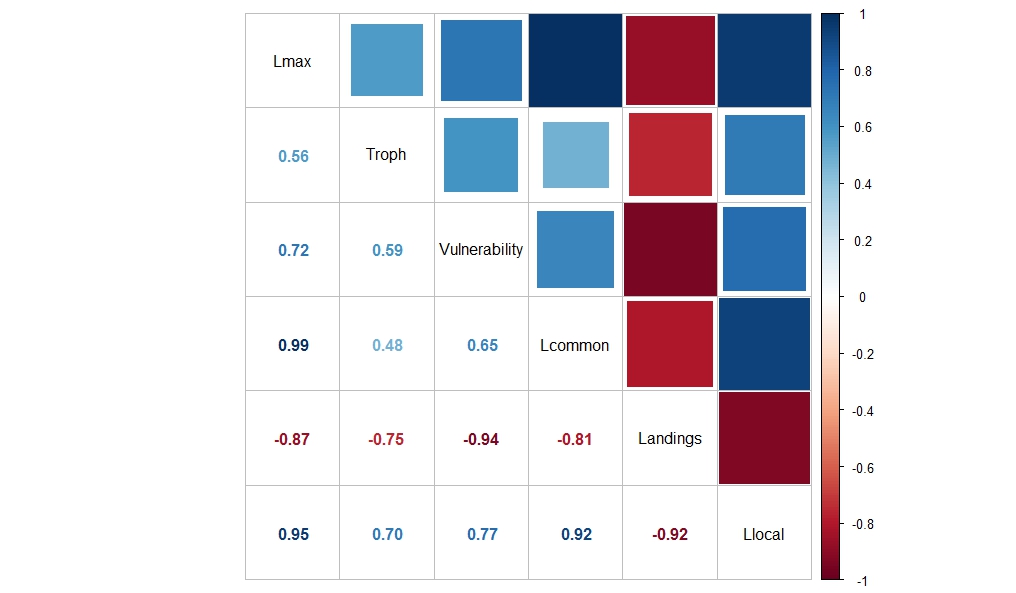


**Figure S5.** Results of correlation analysis of GAM modeling predictors


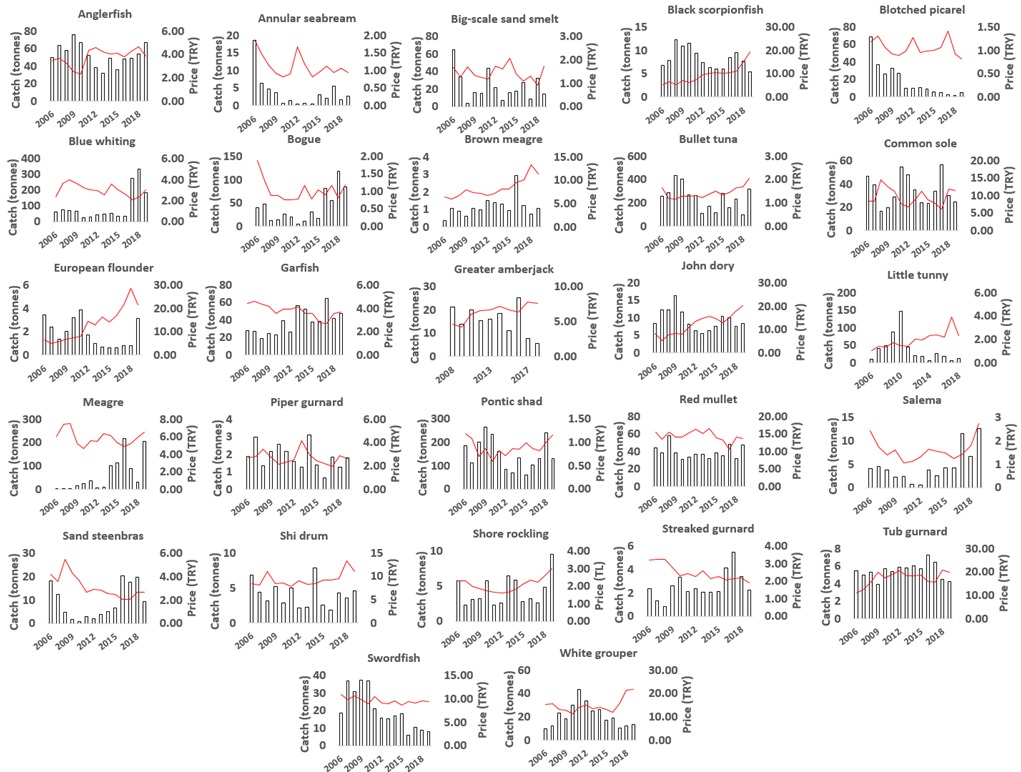


**Figure S6**. Annual changes for 27 species in landed catch (bars) and unit market prices (red lines) for the main commercial fish species between 2006 and 2019 in Istanbul Fish Market.


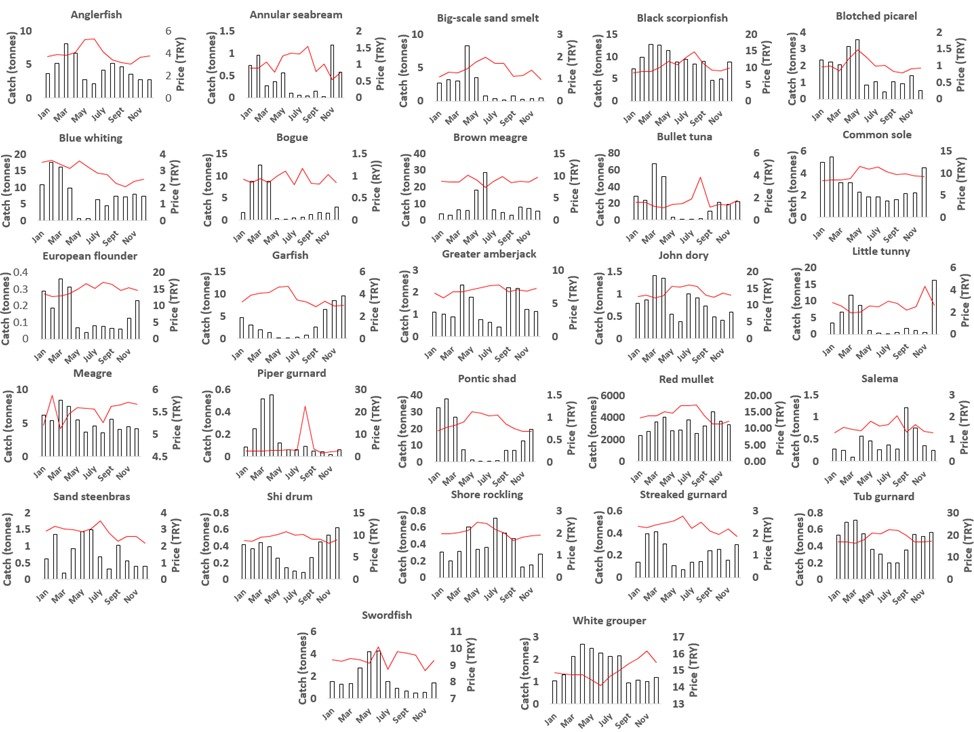


**Figure S7**. Average monthly changes of 27 species in landed catch (bars) and unit prices (red lines) for the key fish species between 2009 and 2019 in Istanbul Fish Market.

**Table S1.** Examined fish species by scientific name, common name, group (SP= small pelagic, MP= medium pelagic, D=Demersal fish), trophic level (TL), vulnerability category (V), maximum recorded length (Lmax), catch length (Lc) with respective references (FishBase, 2020)

| **Species** | **FishBase Data** | | | | **IFM Data** | | **MAFA Data** | **Literature Data** | | |
| --- | --- | --- | --- | --- | --- | --- | --- | --- | --- | --- |
|  | **Lmax** | **Troph** | **Vulnerability** | **Common length** | **Landings** | **Real Price** | **MLS** | **Local Lmax** | **Source of Local Lmax** | **Region** |
| *Alosa immaculata* | 60 | 4 | 50 | 40 | 150.95 | 0.89 |  | 32.8 | Akgümüş, 2017 | Black Sea |
| *Argyrosomus regius* | 230 | 4.3 | 67 | 150 | 60.95 | 5.88 | 25 | 131 | Tokaç et al,2017 | Aegean Sea |
| *Atherina boyeri* | 20 | 3.2 | 44 |  | 23 | 1.45 |  | 13.5 | Gençoğlu, 2017 | Marmara |
| *Auxis rochei* | 50 | 4.4 | 27 |  | 243 | 1.44 |  | 48 | Kahraman et al., 2010 | Mediterranean |
| *Belone belone* | 104 | 4.2 | 49 | 45 | 37.56 | 3.78 |  | 65.1 | Bilgin et al., 2014 | Black Sea |
| *Boops boops* | 40 | 2.8 | 50 | 20 | 40.19 | 1.02 |  | 27.6 | Cengiz et al., 2019 | Aegean Sea |
| *Chelidonichthys lastoviza* | 40 | 3.5 | 32 | 15 | 2.60 | 2.48 |  | 22.1 | Uçkun. 2005 | Aegean Sea |
| *Chelidonichthys lucerna* | 75.1 | 4 | 40 | 30 | 5.48 | 17.41 | 18 | 64 | Bök et al., 2011 | Marmara |
| *Diplodus annularis* | 27.5 | 3.6 | 27 | 13 | 3.72 | 1.14 |  | 16.7 | Demirel and Murat-Dalkara, 2012 | Marmara |
| *Engraulis encrasicolus* | 20 | 3.1 | 25 | 13.5 | 15844 | 1.47 | 9 | 15.2 | Doğu 2049 | Marmara |
| *Epinephelus aeneus* | 120 | 4 | 52 | 60 | 21.06 | 14.95 | 50 | 101 | Özgür Özbek et al., 2013 | Mediterranean |
| *Euthynnus alletteratus* | 122 | 4.5 | 57 | 80 | 37.12 | 1.95 |  | 87 | Kahraman et al., 2008 | Mediterranean |
| *Gaidropsarus mediterraneus* | 50 | 3.5 | 26 |  | 4.30 | 2.09 |  | 25 | Osma, 2019 | Black Sea |
| *Lithognathus mormyrus* | 55 | 3.4 | 40 | 30 | 9.01 | 3.16 |  | 30 | Aydın 2018 | Black Sea |
| *Lophius budegassa* | 100 | 4.4 | 69 | 50 | 52 | 3.81 |  | 107 | Yiğin et al., 2015 | Aegean Sea |
| *Merlangius merlangus* | 70 | 4.4 | 37 | 23 | 2093 | 1.24 | 13 | 41.2 | Bal, 2021 | Marmara |
| *Merluccius merluccius* | 140 | 4.4 | 64 | 45 | 369 | 3.91 |  | 58.8 | Yildiz, 2020 | Marmara |
| *Micromesistius poutassou* | 55 | 4.1 | 34 | 20 | 95 | 3.02 |  | 39 | İşmen et al. 2010 | Aegean Sea |
| *Mullus barbatus* | 33.2 | 3.1 | 29 | 20 | 39 | 14.39 | 13 | 22.7 | Demirel and Murat-Dalkara, 2012 | Marmara |
| *Mullus surmuletus* | 40 | 3.5 | 39 | 25 | 867 | 5.76 | 11 | 23.5 | Moldur, 1999 | Marmara |
| *Pleuronectes flesus* | 100 | 3.2 | 71 | 40 | 1.84 | 13.28 | 20 | 37.5 | Şahin and Güneş, 1998 | Black Sea |
| *Pomatomus saltatrix* | 130 | 4.5 | 63 | 60 | 2301 | 12.80 | 18 | 86 | Türgan, 1959 | Marmara |
| *Sarda sarda* | 91.4 | 4.5 | 33 | 50 | 3768 | 6.86 | 25 | 63 | Kahraman et al., 2014 | Marmara |
| *Sardina pilchardus* | 27.5 | 3.1 | 27 | 20 | 860 | 1.13 | 11 | 14.3 | Erdoğan et al., 2010 | Edremit Bay |
| *Sarpa salpa* | 51 | 2 | 41 | 30 | 4.57 | 1.6 |  | 33.1 | Bektaş, 2017 | North Aegean |
| *Sciaena umbra* | 70 | 3.8 | 64 | 28 | 1.14 | 8.27 | 35 | 63 | Engin and Seyhan, 2009 | Black Sea |
| *Scomber colias* | 64 | 3.4 | 31 | 30 | 301 | 1.97 | 18 | 31.1 | Cengiz, 2012 | Aegean Sea |
| *Scomber scombrus* | 60 | 3.6 | 44 | 30 | 82.77 | 4.94 | 20 | 27 | Bal and Türker | Marmara |
| *Scopthalmus maximus* | 100 | 4.4 | 43 | 50 | 138 | 19.56 | 45 | 70 | Erylmaz and Dalyan, 2015 | Black Sea |
| *Scorpaena porcus* | 40.5 | 3.9 | 51 | 15 | 107 | 9.56 | 18 | 32 | Alparslan et al., 2007 | Marmara |
| *Seriola dumerili* | 190 | 4.5 | 54 | 100 | 15.41 | 6.37 | 30 | 142 | Tuncer et al., 2010 | Marmara |
| *Solea solea* | 70 | 3.2 | 36 | 35 | 34 | 9.73 | 20 | 34 | Oral, 1996 | Marmara |
| *Spicara maena* | 20 | 3 | 39 | 14 | 18.15 | 1.05 |  | 18.8 | Sevgili et al., 2016 | Marmara |
| *Trachurus mediterraneus* | 60 | 3.8 | 47 | 30 | 5094 | 2.15 | 13 | 18.5 | Demirel and Murat-Dalkara, 2012 | Marmara |
| *Trachurus trachurus* | 70 | 3.7 | 59 | 22 | 635 | 1.10 | 13 | 21 | Demirel and Murat-Dalkara, 2012 | Marmara |
| *Trigla lyra* | 60 | 3.7 | 63 | 30 | 1.85 | 2.71 |  | 51 | Bök et al., 2011 | Marmara |
| *Umbrina cirrosa* | 73 | 3.4 | 40 | 40 | 4.02 | 9.29 | 45 | 94 | Aydın and Sözer, 2020 | Black Sea |
| *Xiphias gladius* | 455 | 4.5 | 72 | 300 | 20 | 9.62 | 125 | 161 | Alıçlı et al 2012 | Mediterranean |
| *Zeus faber* | 90 | 4.5 | 68 | 40 | 9.43 | 12.40 |  | 52.8 | İşmen et al. 2012 | Aegean Sea |

**Table S2.** Different market sizes of bonito and bluefish in Turkey.

| **Fish** | **TL (cm)** | **TW (g)** | **Acknowledged Turkish name** |
| --- | --- | --- | --- |
| Bluefish | 25 - 35 | 250 - 500 | Lüfer |
|  | 35 - 40; >40 | 1000 - 1500; > 1500 | Kofana |
| Atlantic bonito | 30 - 35 | 600 -900 | Palamut |
|  | 50-60 | 3000-4000 | Torik |
